# Therapeutic value of mesenchymal stem cell-derived extracellular vesicles in hypertrophic and keloid scars: a systematic review and meta-analysis

**DOI:** 10.3389/fcell.2026.1739106

**Published:** 2026-01-28

**Authors:** Tianhui Zhai, Wanqi Tang, Pengchao Liu, Yakun Liang, Zhihong Ma, Leiqiang Fan

**Affiliations:** 1 Department of Plastic Surgery, Chengde Central Hospital, Chengde Medical College, Chengde, Hebei, China; 2 School of Basic Medicine, Tongji Medical College, Huazhong University of Science and Technology, Wuhan, China; 3 Department of Urology, Beijing Nuclear Industry Hospital, Beijing, China; 4 Department of Plastic Surgery, Chengde Central Hospital, Chengde, China; 5 Department of Dermatology, Chengde Central Hospital, Chengde, China

**Keywords:** collagen remodeling, extracellular vesicles, fibrosis, mesenchymal stem cell, SCAR

## Abstract

**Background:**

Keloids and hypertrophic scars are pathological wound healing responses characterized by excessive scar tissue formation, presenting significant challenges to both patients and healthcare systems globally. Existing evidence demonstrates that mesenchymal stem cell–derived extracellular vesicles (MSC-EVs) can attenuate collagen deposition and contraction in scar tissue; however, their application in the treatment of hypertrophic scars and keloids remains largely at the preclinical stage. This systematic review aims to critically assess preclinical studies on the therapeutic efficacy of MSC-EVs in the management of keloids and hypertrophic scars. The review synthesizes findings from controlled and interventional studies, focusing on the use of MSC-EVs in animal models of these scars and their application in human subjects with raised scars following skin injury.

**Methods:**

A total of 15 studies involving 253 animals were identified through a comprehensive search of the PubMed, Cochrane, Embase, MEDLINE Complete, Web of Science, CNKI, and Wanfang databases, covering the period from their inception to August 29, 2025. The aim was to evaluate the effects of MSC-EV therapy on keloids and hypertrophic scars through a meta-analysis of the standardized mean difference (SMD) in preclinical animal models. Meta-analyses were conducted using Stata 18 software.

**Results:**

Meta-analysis indicated that compared with the control group, MSC-Exos treatment group can significantly reduce. The dimensions of hypertrophic scars and keloids [(SMD) −2.78, 95% confidence interval (CI) −3.88–1.69)]. Also attenuate other outcomes, such as Collagen Type I [SMD = −4.39, 95%CI: −5.96–2.81], Collagen Type III [SMD = −5.19, 95%CI: −6.93–3.44], migration and proliferation of skin fibroblasts, and the expression of Transforming Growth Factor-β1 (TGF-β1) and α-smooth muscle actin (α-SMA) in scar tissue.

**Conclusion:**

The meta-analysis supports the therapeutic potential of MSC-EVs in the treatment of keloids and hypertrophic scars, as demonstrated in preclinical animal models. MSC-EV therapy has been shown to downregulate the dimensions of hypertrophic scars and keloids, inhibit collagen deposition, and reduce migration and proliferation of skin fibroblasts. Additionally, MSC-EVs suppress the expression of TGF-β1 and α-SMA in scar tissue. These findings highlight MSC-EVs as a promising therapeutic approach for managing keloids and hypertrophic scars.

## 1 Introduction

Keloids and hypertrophic scars are elevated, fibrous lesions that result from abnormal wound healing after skin injury. These scars arise primarily due to prolonged inflammation within the reticular dermis, which drives pathological tissue remodeling. When the skin is injured or exposed to various stimuli, the wound typically progresses through three distinct yet overlapping phases of healing: hemostasis/inflammation, proliferation, and remodeling. These phases unfold in a temporal sequence, with some overlap, and any disruption during these stages can lead to scars that extend beyond the normal skin surface (Murakami and Shigeki, 2024; Ogawa, 2017) Clinically, hypertrophic scars are characterized by the presence of fibrous tissue that remains confined within the boundaries of the original wound, whereas keloids are distinguished by the proliferation of scar tissue that extends beyond the wound margins into the surrounding normal skin. Histologically, the distinction between hypertrophic scars and keloids is primarily quantitative, rather than qualitative, as both exhibit similar pathological features. Specifically, overexpression of Transforming Growth Factor-β1/β2 (TGF-β1/β2), α-smooth muscle actin (α-SMA), and collagen is a hallmark of both lesion types (Limandjaja et al., 2021). The aesthetic and functional consequences of keloids and hypertrophic scars—such as pain, pruritus, and impaired mobility—can significantly impact a patient’s quality of life (Gauglitz et al., 2011). Given these challenges, the global market for scar treatment is projected to reach $32 billion by 2027 (Sen, 2021).

The development of keloids and hypertrophic scars is influenced by a combination of local factors (e.g., wound or scar tension), systemic factors (e.g., hypertension, pregnancy), and genetic factors (e.g., sex, single nucleotide polymorphisms, skin pigmentation). External factors, such as the consumption of hot and spicy foods or exposure to hot showers, have also been implicated in the varying incidence rates of these conditions (Murakami and Shigeki, 2024). Despite the identification of these contributing factors, the exact pathogenic mechanisms remain unclear, and the clinical course is unpredictable. Even with treatment, recurrence rates remain high, preventing the establishment of a universally accepted gold standard for therapy. Conventional management strategies include the use of silicone-based dressings, intralesional corticosteroid injections, radiotherapy, laser therapy, cryotherapy, surgical excision, and various intralesional agents, such as triamcinolone, 5-fluorouracil, verapamil, and botulinum toxin A (Kim et al., 2013; Ekstein et al., 2021; Ogawa, 2022; Yuan B. et al., 2023). Silicone dressings are commonly used as both a preventive and therapeutic approach, with evidence supporting their effectiveness in reducing scar height, erythema, and pruritus. Surgical excision is typically indicated for larger or refractory scars but is associated with a high recurrence rate unless combined with adjunctive therapies. Cryotherapy has proven effective in reducing scar volume and recurrence rates, though it carries a risk of hypopigmentation, particularly in individuals with darker skin tones. Intralesional injections offer targeted delivery of therapeutic agents directly to scar tissue, addressing the underlying pathological mechanisms to reduce scar size, alleviate symptoms, and decrease recurrence. Corticosteroid injections, radiotherapy, and laser therapy are often used as adjunctive treatments and typically require multiple administrations for optimal results. Although current therapeutic strategies primarily aim to reduce scar volume and alleviate symptoms, their efficacy and adverse effects exhibit significant variability. Advances in understanding the molecular mechanisms of keloid and hypertrophic scar formation have led to the development of novel therapies targeting specific signaling pathways and genes. These emerging treatments hold promise for more precise, personalized therapies tailored to the molecular profile of each scar, potentially improving therapeutic outcomes (Kim and Kim, 2024).

Mesenchymal stem cells (MSCs) are multipotent adult stem cells with the ability to proliferate, self-renew, and differentiate into various cellular lineages. Found in nearly all postnatal organs and tissues, MSC-based therapies have demonstrated significant therapeutic potential for a broad spectrum of diseases (Hade et al., 2021; da Silva Meirelles et al., 2006). Recent research increasingly highlights that MSC-mediated repair primarily depends on the secretion of paracrine factors and vesicles, including exosomes. Exosomes, a key form of cell-free therapy, have shown therapeutic benefits in numerous conditions, including neurological, pulmonary, cartilage, renal, cardiac, and hepatic diseases, as well as in bone regeneration and cancer, based on preclinical animal and cellular studies (Hade et al., 2021; Yin et al., 2019; Barreca et al., 2020). These extracellular vesicles (EVs) function as efficient delivery vehicles, capable of encapsulating and transporting biologically active molecules such as proteins, peptides, nucleic acids, and lipids (Théry et al., 2002). Through the facilitation of exogenous compound and biomolecule transport, exosomes have become a valuable tool in cell-free regenerative medicine. Compared to traditional cell-based therapies, cell-free formulations offer several advantages, including improved accessibility, lower costs, and enhanced storage and distribution capabilities. These benefits position exosome-based therapies as a promising alternative in regenerative medicine (Montero-Vilchez et al., 2021).

EVs, nanosized particles secreted by nearly all cell types, are encased in a lipid bilayer. Based on their size and cellular origin, EVs are classified into three main types: exosomes (50–150 nm), microvesicles (MVs, 100–1000 nm), and apoptotic bodies (500–5000 nm) (Kee et al., 2022; Qiu et al., 2019). MSC-derived nanoscale vesicles are emerging as a promising therapeutic strategy for wound repair; however, their application in treating keloids and hypertrophic scars remains confined to preclinical studies, with their efficacy yet to be definitively established (Huang et al., 2021; Casado-Díaz et al., 2020). Exosomes regulate scar formation by inhibiting fibroblast proliferation and migration, preventing sustained activation into myofibroblasts, and reducing the excessive synthesis and deposition of extracellular matrix (ECM) proteins, particularly COL1 and COL3 (Kim and Kim, 2024). Topically applied EVs exhibit limited skin penetration, primarily reaching the stratum corneum, with less than 1% permeating into the granular layer (Kee et al., 2022; Zhang et al., 2021). To address this limitation, researchers have investigated integrating EVs with physical and chemical permeation-enhancing technologies to improve skin penetration and maximize therapeutic efficacy (Park and Yi, 2024). Physical strategies, such as microneedles, facilitate transdermal drug delivery and are recognized for their safety, painlessness, convenience, and minimal invasiveness (Hao et al., 2017). Current clinical studies suggest that combining exosome preparations with microneedle-based transdermal delivery systems offers therapeutic benefits in managing established keloids. Chemical enhancement strategies typically involve hydrogel- or biomaterial-based dressings, with hydrogels being commonly employed for controlled drug release. Their adjustable release kinetics help counteract the short half-life of EVs, thereby improving therapeutic efficacy. Shen et al. developed a double-layer thiolated alginate/poly (ethylene glycol) diacrylate hydrogel designed for the sequential delivery of two types of EVs: BMSC-derived EVs in the upper layer and miR-29b-3p-loaded EVs in the lower layer. This innovative approach significantly enhanced full-thickness wound healing in rat and rabbit ear models. The bilayer SA-SH/PEG-DA hydrogel facilitates the sequential release of EVs, promoting accelerated wound closure in the early stages of healing while preventing hypertrophic scar formation in the later stages (Shen et al., 2021).

In summary, based on the current state of EV research and their promising clinical translational potential, we are confident in the therapeutic prospects of EVs for the treatment of keloids and hypertrophic scars. Nevertheless, although mesenchymal stem cell–derived extracellular vesicles (MSC-EVs) represent a highly promising alternative therapeutic strategy for these fibrotic skin disorders, further evidence is reqMSCed to substantiate the feasibility of their large-scale production, commercialization, and clinical efficacy. Therefore, this study aims to systematically consolidate findings from multiple sources of MSC-EVs to evaluate their therapeutic effects and to perform a meta-analysis of available preclinical studies, thereby providing a more comprehensive theoretical foundation for their subsequent clinical translation.

## 2 Methods

### 2.1 Protocol

The protocol for this study was following the guidance in the updated Pre)ferred Reporting Items for Systematic Reviews and Meta-analyses (PRISMA) (Page et al., 2021). The study’s protocol was registered on PROSPERO(CRD420251080087)

### 2.2 Literature search strategy

Literature search strategy We searched PubMed, Cochrane, MEDLINE Complete, Web of Science databases, Embase, CNKI and Wanfang data for articles published without restriction on publication dates to 29 August 2025 With the combination of subject words and free words, the search terms included three categories: (1) “mesenchymal stromal cell”, “multipotent stromal cell”, “adipose-Derived Stem Cell”, and “bone mesenchymal stem cell”; and (2) “exosome”, “extracellular vesicle”, and “microparticle”; and (3) “keloid”, “hypertrophic scar”, and “pathologic scar”. The logical relationship was created with “OR” and “AND”; and the search formula was thereafter developed according to the characteristics of the different databases (supplementary data). The search was limited to animal trial studies. A preretrieval process improved the searches strategy. In addition, we performed a manual search of the references of the studies to obtain further potential studies. For the analysis we included studies reported in only English.

### 2.3 Inclusion criteria

The inclusion criteria for studies were as follows:

1. Utilization of *in vivo* and *in vitro* experimental animal models.
2. The experimental group received mesenchymal stem cell-derived extracellular vesicle (MSC-Exo) therapy.
3. The control group received either a non-functional solution or no treatment.
4. The study subjects involved keloids or hypertrophic scars.

### 2.4 Exclusion criteria

Studies were excluded based on the following conditions:

1. Use of plant-derived EVs or EVs from sources other than mesenchymal stem cells.
2. Studies involving *in vivo* and *in vitro* models of conditions other than keloids or hypertrophic scars.
3. Randomized or non-randomized clinical trials.
4. Studies using animal models other than rabbits, mice, or rats.
5. Studies published in languages other than English.
6. Studies that were theses, conference abstracts, or review articles.

### 2.5 Study selection process

All identified citations were imported into Zotero for systematic management of the search records, after the removal of duplicates. Two independent researchers screened the titles and abstracts of the studies to exclude those that did not meet the predefined inclusion criteria. As an additional precaution, the full texts of potentially relevant studies were assessed for final eligibility. Any disagreements between the two reviewers were resolved through discussion with a third team member. The study selection process was summarized in a flow diagram, in accordance with the PRISMA guidelines.

### 2.6 Data extraction

Relevant data were independently extracted by two reviewers from the included studies using a standardized, pilot-tested data extraction form in Excel (Microsoft, Seattle, United States). In cases where raw data were not provided, data were extracted from graphical representations using WebPlotDigitizer4.8. The following data were retrieved: 1) Study characteristics, including authors, study type, publication year, and country of origin; 2) Study population details, such as sample size, group, species, and scar size and depth; 3) Intervention characteristics, including criteria for MSC and EV characterization, MSC source, MSC-EV dose, and route of administration; 4) Study design information, such as comparator, sample size, MSC-EV isolation methods, and characterization protocols; 5) Measurement indicators, including scar size, cellular behaviors, collagen deposition, and protein levels of TGF-β1 and α-SMA; and 6) Risk of bias details.

### 2.7 Literature quality evaluation

STAIR was used to assess the quality of the included studies (Fisher et al., 2009). The items were as follows: (1) Sample size calculation. (2) Inclusion and exclusion criteria. (3) Randomisation. (4) Allocation concealment. (5) Reporting of animals excluded from the analysis. (6) Patients were blinded to the assessment of outcome. (7) Reporting potential conflicts of interest and study funding. Rate “Yes”, “No” or “Unclear”.

### 2.8 Statistical analysis

Data were analyzed using Stata 18 (Stata LP, Texas, United States). All results were treated as continuous variables and expressed as SMDs with 95% confidence intervals (CIs). A P value of <0.05 was considered indicative of a significant difference between the experimental and control groups, reflecting a substantial effect size (Higgins and Thompson, 2002). A fixed-effects model was utilized to quantitatively synthesize each outcome measure. In the presence of significant between-study heterogeneity, random-effects models were applied. The Cochrane Q test was employed to assess heterogeneity among studies, and the I^2^ statistic was used to quantify the extent of this heterogeneity. A value greater than 50% was interpreted as substantial heterogeneity. When high heterogeneity was detected, the DerSimonian-Laird random-effects model (DL model) was used to estimate the average effect size and its corresponding confidence interval. A sensitivity analysis was conducted using Stata 18 to compare the new combined effect size with the original combined effect size, in order to determine if the results had changed substantially. If the effect size corresponding to an individual study fell outside the 95% CIs, the exclusion of that study was assessed for its impact on the total combined effect size. Additionally, if the combined effect size after excluding a study significantly differed from the combined effect size including all studies, the results were considered to be influenced by that study.

## 3 Results

### 3.1 Study selection

**FIGURE 1:**
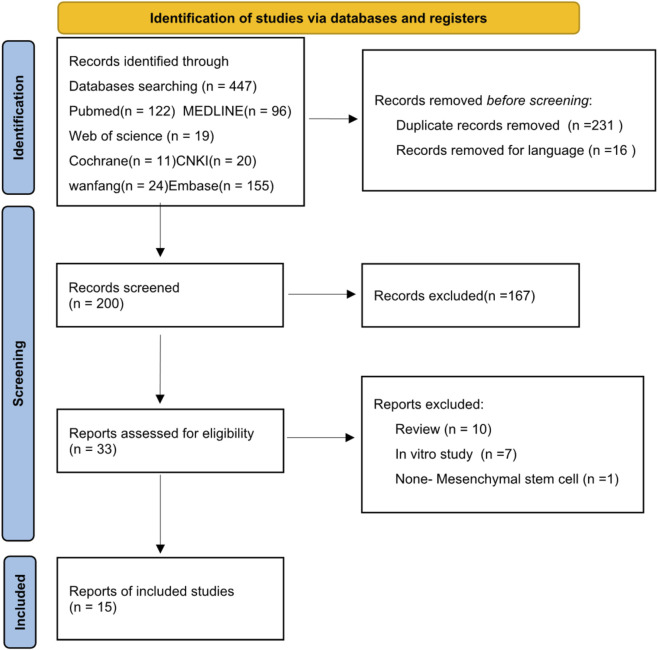
Flowchart of the study selection process.

A total of 447 articles were initially screened. After removing duplicates and excluding Chinese-language publications, 200 papers were retained. Following a thorough screening of titles, abstracts, and full texts (Figure 1), 15 studies involving a total of 253 animals were ultimately included (Chen et al., 2023; Jiang et al., 2020; Liu et al., 2024; Li et al., 2021; Meng et al., 2024; Tian et al., 2024; Wang et al., 2025; Xu Z. et al., 2024; Xu C. et al., 2024; Ye et al., 2025; Yuan et al., 2021; Zhao et al., 2025; Zhen et al., 2025; Zhu et al., 2024; Zhu et al., 2020).

### 3.2 Characteristics of the studies considered for use in analysis

The baseline characteristics and methodological quality assessment results of these studies are summarized in Table 1 (Characteristics of the included studies) and Table 2 (Quality assessment). Table 1 outlines the characteristics of the 15 included studies, which were published between 2020 and 2025, with an aggregate sample size of 253. Of these, eight studies (Chen et al., 2023; Jiang et al., 2020; Liu et al., 2024; Li et al., 2021; Tian et al., 2024; Xu C. et al., 2024; Yuan et al., 2021; Zhu et al., 2024) utilized mice, two (Zhao et al., 2025; Zhen et al., 2025) employed rats and five (Meng et al., 2024; Wang et al., 2025; Xu Z. et al., 2024; Ye et al., 2025; Zhu et al., 2020) used rabbit as animal models. In terms of tissue sources, ten studies (Chen et al., 2023; Liu et al., 2024; Li et al., 2021; Meng et al., 2024; Tian et al., 2024; Wang et al., 2025; Xu Z. et al., 2024; Xu C. et al., 2024; Yuan et al., 2021; Zhu et al., 2020) used human adipose tissue, two (Zhao et al., 2025; Zhen et al., 2025) used human epidermal tissue, two (Jiang et al., 2020; Zhu et al., 2024) used human bone marrow tissue, and one (Ye et al., 2025) study utilized human umbilical cord tissue. All included studies were conducted in China. Exosome isolation and validation in these studies were performed using several methods, including ultracentrifugation for exosome extraction and identification, transmission electron microscopy (TEM) for morphological assessment of adipose-derived mesenchymal stem cells exosomes (ADSC-Exos), nanoparticle tracking analysis (NTA) for determining particle size distribution, and Western blotting to detect membrane surface markers such as Alix, TSG101, CD9, CD63, and CD81.All studies employed rodent models with full-thickness skin defects, ranging from 6 to 20 mm in diameter, located on the back or footpad. The control groups in the studies included various types of controls, such as phosphate-buffered saline and placebo treatments. Significant heterogeneity was observed across the intervention and control treatments for all outcome variables (P < 0.05).

**TABLE 1:** Characteristics of the included studies.

First author	Year	Country	Animal (number)	RCT	MSCs source	Wound/Keloid	Positive surface markers		Negative surface markers		Method
							Stem cell markers	Exosomes makers	Stem cell markers	Exosomes makers	
Chen	2023	China	BABL/c mice (8)	NA	Human adipose tissue	1 × 1 cm	CD29,CD90	CD9,CD63	CD34,CD45	Not described	Subcutaneous injection,100 μg/100 μL (5 μg/g bodyweight)
Jiang	2020	China	C57BL/6J mice (36)	RCT	Human bone marrow	Diameter 6 mm	Not described	TSG101,CD9,CD63,Alix	Not described	Not described	Subcutaneous injection,100 μg/100 μL
Liu	2024	China	BALB/c mice (15)	RCT	Human adipose tissue	Not described	CD105,CD90,CD29	CD9,TSG101,CD63	CD45,CD30	Not described	Subcutaneous injection,50 μL
Li	2021	China	BABL/c mice (12)	RCT	Human adipose tissue	1 × 1 cm	CD29,CD44,CD73,CD90	CD63,CD9	CD34,CD45	Cd68	Subcutaneous injection,70 μg/100 μL
Meng	2023	China	New Zealand rabbits (18)	RCT	Human adipose tissue	1 × 1 cm	Not described	CD9,TSG101,CD63	Not described	Calnexin	Subcutaneous injection,4 × 10^8^ particles/wound
Tian	2024	China	nude mice (24)	NA	Human adipose tissue	1 × 1 × 1 cm	CD29,CD44,CD105	CD9,TSG101,CD63	CD45,CD14,CD45	Not described	Subcutaneous injection,200 μg/200 mL
Wang	2025	China	New Zealand rabbits (9)	RCT	Human adipose tissue	10 × 10 mm	Not described	Not described	Not described	Not described	GelMA hydrogels
Xu-1	2024	China	New Zealand rabbits (6)	RCT	Human adipose tissue	0.1 × 0.1 mm	Not described	Not described	Not described	Not described	Subcutaneous injection,100 μg/2 mL
Xu-2	2024	China	BABL/c mice (12)	RCT	Human adipose tissue	1 x 1 cm	CD29,CD44,CD73,CD90	CD9,TSG101,CD63	CD34,CD45	Calnexin	Subcutaneous injection,100 μg/100 μL
Ye	2025	China	New Zealand rabbits (1)	RCT	Human umbilical cord mesenchymal	Diameter 1 cm	CD105, CD29, CD44	CD9,TSG101,CD63	HLA-DR,CD34,CD45	Not described	Subcutaneous injection,200 μg/200 μL
Yuan	2021	China	Kunming mice (36)	RCT	Human adipose tissue	Diameter 2 cm	Not described	TSG101,CD81,CD63	Not described	Not described	Subcutaneous injection,30 mg/kg
Zhao	2025	China	SD rats (36)	RCT	Human epidermal tissue	8 × 8 mm	ITGα6	CD81,TSG101	CD71,K10	Calnexin	Subcutaneous injection,500 μg/mL
Zhen	2025	China	Sprague–Dawley rats (12)	RCT	Human epidermal tissue	6 × 6 mm	cd49f,Krt15	Alix,TSG101,CD81,CD63	Not described	Not described	Subcutaneous injection,10 μg/200 μL
Zhu	2024	China	NOD SCID mice (12)	NA	Human bone marrow	4 × 4 × 4 mm	Not described	CD9,CD63,CD81	Not described	Calnexin	Subcutaneous injection,50 μg/100 μL
Zhu	2020	China	New Zealand rabbits (16)	RCT	Human adipose tissue	Diameter 8 mm	CD105,CD90,CD73,CD49d	CD63,TSG101,Alix	CD34,CD45	Not described	Subcutaneous injection,0.1 mL

NA, not available; RCTs, randomised controlled trials; ADSCs, adipose-derived stem cells.

**TABLE 2:** Literature quality evaluation.

Study	A	B	C	D	E	F	G
Chen	Yes	Unclear	Unclear	No	Unclear	Unclear	Yes
Jiang	Yes	Unclear	Unclear	No	Unclear	Unclear	Yes
Liu	Yes	Unclear	Unclear	No	Unclear	Unclear	Yes
Li	Yes	Unclear	Unclear	No	Unclear	Unclear	Yes
Meng	Yes	Unclear	Unclear	No	Unclear	Unclear	Yes
Tian	Yes	Unclear	Unclear	No	Unclear	Unclear	Yes
Wang	Yes	Unclear	Unclear	No	Unclear	Unclear	Yes
Xu-1	Yes	Unclear	Unclear	No	Unclear	Unclear	Yes
Xu-2	Yes	Unclear	Unclear	No	Unclear	Unclear	Yes
Ye	Yes	Unclear	Unclear	No	Unclear	Unclear	Yes
Yuan	Yes	Unclear	Unclear	No	Unclear	Unclear	Yes
Zhao	Yes	Unclear	Unclear	No	Unclear	Unclear	Yes
Zhen	Yes	Unclear	Unclear	No	Unclear	Unclear	Yes
Zhu	Yes	Unclear	Unclear	No	Unclear	Unclear	Yes
Zhu	Yes	Unclear	Unclear	No	Unclear	Unclear	Yes

A Sample size calculation, B inclusion and exclusion criteria, C randomisation, D allocation concealment, E reporting of animals excluded from the analysis, F blinded assessment of outcome, G reporting potential conflicts of interest and study funding.

### 3.3 Quality assessment

The Systematic Review of Animal Intervention Studies (STAIR) tool was utilized to evaluate the methodological quality of the included studies. The majority of the studies were randomized controlled trials (RCTs); however, the specific randomization methods employed were not clearly specified. Several studies either failed to report the use of randomization or did not mention allocation concealment. A detailed summary of the study quality assessment is presented in Table 2.

### 3.4 Synthesized findings

#### 3.4.1 Primary outcome

**FIGURE 2:**
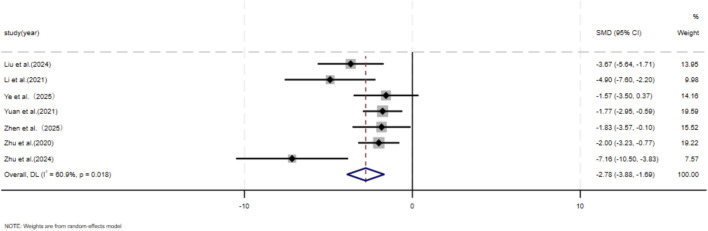
The forest plot visually illustrates the efficacy of exosome therapy in promoting scar reduction, where negative SMD values represent enhanced scar reduction rates, DL Der simonian Laird Random effects method, CI confidence interval, SMD standard mean difference.

The dimensions of hypertrophic scars and keloids. A comprehensive analysis encompassing seven independent studies (Liu et al., 2024; Li et al., 2021; Ye et al., 2025; Yuan et al., 2021; Zhen et al., 2025; Zhu et al., 2024; Zhu et al., 2020) encompassed 80 animal models, evenly distributed between an experimental group (n = 40) and a control group (n = 40), to document scar reduction rates. Given substantial heterogeneity between studies (p < 0.05, I^2^ > 50%), DL model was employed for assessment. The meta-analysis results unequivocally demonstrated that, at the final assessment date of the trials, the experimental cohort exhibited a significantly faster rate of scar reduction compared to the control cohort (SMD = −2.78, 95%CI: −3.88–1.69, p = 0.018) (Figure 2).

#### 3.4.2 Secondary outcome

##### 3.4.2.1 Collagen deposition

**FIGURE 3:**
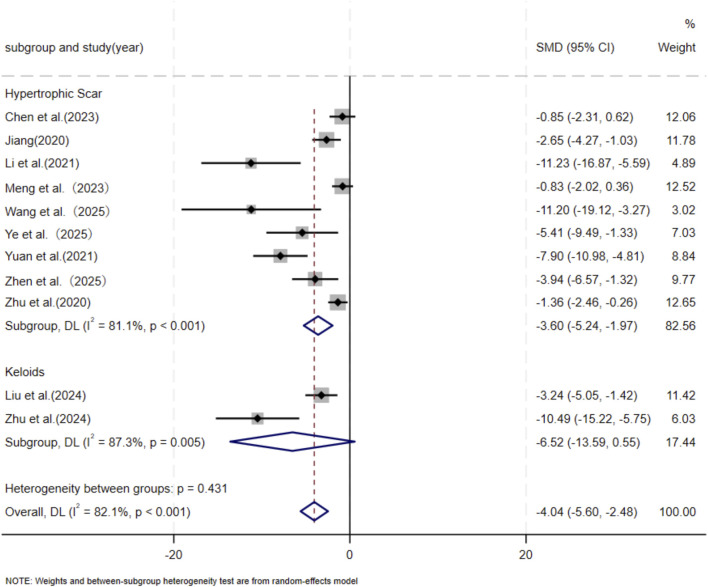
Forest plot visually illustrates the effect of exosome therapy on the proportion of COL1 deposition, where negative SMD values represent reduced COL1 accumulation.*CI* confidence interval, SMD standard mean difference, DL Der simonian Laird Random effects method.

**FIGURE 4:**
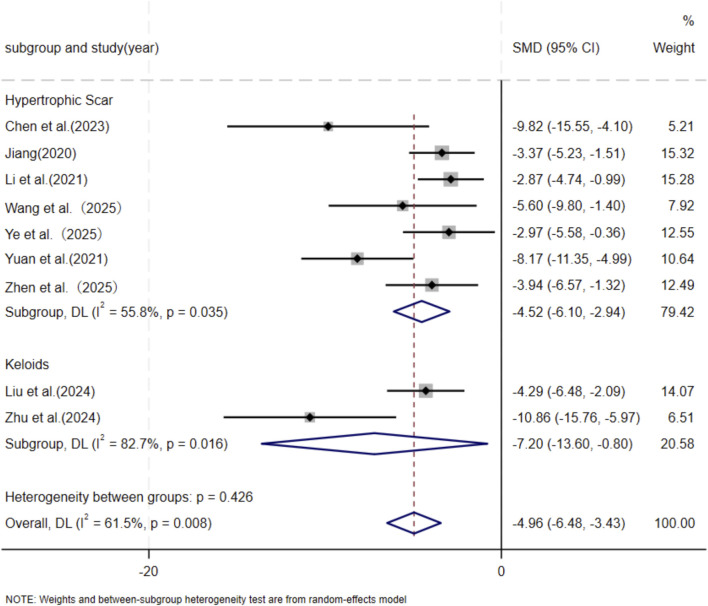
Forest plot visually illustrates the effect of exosome therapy on the proportion of COL3 deposition, where negative SMD values represent reduced COL3 accumulation.CI confidence interval, SMD standard mean difference, DL Der simonian Laird Random effects method.

Eleven independent studies (Chen et al., 2023; Jiang et al., 2020; Liu et al., 2024; Li et al., 2021; Meng et al., 2024; Wang et al., 2025; Ye et al., 2025; Yuan et al., 2021; Zhen et al., 2025; Zhu et al., 2024; Zhu et al., 2020), including 118 animal models equally allocated to experimental and control groups (n = 59 per group), evaluated COL1 deposition in wound tissue. Eight studies (Chen et al., 2023; Jiang et al., 2020; Liu et al., 2024; Li et al., 2021; Wang et al., 2025; Ye et al., 2025; Yuan et al., 2021; Zhu et al., 2024) comprising 82 animal models (n = 41 per group), assessed COL3 deposition. Excessive collagen accumulation is a key pathological feature underlying the raised, erythematous, and rigid lesions observed in hypertrophic scars and keloids. Keloids are typically characterized by increased type I collagen, leading to an elevated type I/type III collagen ratio, whereas hypertrophic scars exhibit a pronounced increase in type III collagen (Friedman et al., 1993; Kohlhauser et al., 2024). Based on these distinct collagen profiles, subgroup analyses were conducted according to scar type. Among studies evaluating COL1 deposition, nine used hypertrophic scar–related models and demonstrated a significant reduction in the MSC-EV–treated group compared with controls (SMD = −3.60, 95% CI: −5.24 to −1.97, *p* < 0.001). Two studies using keloid-related models also showed a significant decrease in COL1 deposition (SMD = −6.52, 95% CI: −13.59 to −0.55, *p* = 0.005). Pooled analysis indicated a significant overall reduction in COL1 deposition in the experimental group relative to controls at the final assessment time point (SMD = −4.04, 95% CI: −5.60 to −2.48, *p* < 0.001) (Figure 3). Regarding COL3 deposition, six studies derived from hypertrophic scar–related models showed a significant reduction following MSC-EV treatment (SMD = −4.73, 95% CI: −6.63 to −2.83, *p* = 0.019), while two keloid-related studies reported a significant decrease (SMD = −7.20, 95% CI: −13.60 to −0.80, *p* = 0.016). The pooled meta-analysis demonstrated a significant overall reduction in COL3 deposition in the experimental group compared with the control group (SMD = −5.19, 95% CI: −6.93 to −3.44, *p* = 0.004) (Figure 4).

##### 3.4.2.2 Migration and proliferation of skin fibroblasts

**FIGURE 5:**
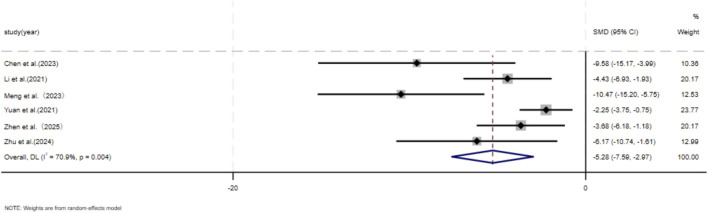
Forest plot visually illustrates the effect of exosome therapy on the migration of skin fibroblasts. CI confidence interval, SMD standard mean difference, DL Der simonian Laird Random effects method.

**FIGURE 6:**
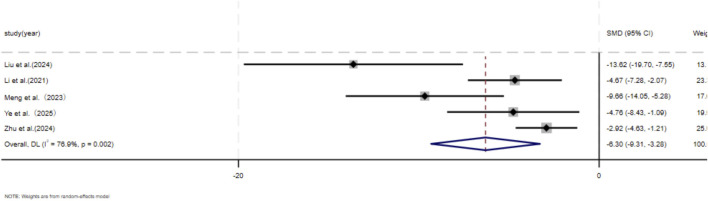
Forest plot visually illustrates the effect of exosome therapy on the proliferation of skin fibroblasts. CI confidence interval, SMD standard mean difference, DL Der simonian Laird Random effects method.

Six independent studies (Chen et al., 2023; Li et al., 2021; Meng et al., 2024; Yuan et al., 2021; Zhen et al., 2025; Zhu et al., 2024) including a total of 56 animal models equally allocated to experimental and control groups (n = 28 per group), evaluated skin fibroblast migration. In addition, five studies (Liu et al., 2024; Li et al., 2021; Meng et al., 2024; Ye et al., 2025; Zhu et al., 2024) comprising 52 animal models (n = 26 per group), assessed skin fibroblast proliferation. In response to wound-associated stimuli, fibroblasts exhibit enhanced proliferative and migratory capacities and resistance to apoptosis (Ma et al., 2024). Accordingly, several studies have identified fibroblast migratory ability as a core cellular characteristic in the wound-healing and scarring process (Sadiq et al., 2024). Given the substantial heterogeneity observed among studies (*p* < 0.05, I^2^ > 50%), a DerSimonian–Laird random-effects model was applied for meta-analysis. At the final assessment time point, pooled results demonstrated a significant reduction in fibroblast migration in the experimental group compared with the control group (SMD = −5.28, 95% CI: −7.59 to −2.97, p = 0.004) (Figure 5). Similarly, MSC-EV treatment resulted in a significant decrease in fibroblast proliferation relative to controls (SMD = −6.29, 95% CI: −9.31 to −3.28, p = 0.004) (Figure 6).

##### 3.4.2.3 Expression of α-SMA and TGF-β1

**FIGURE 7:**
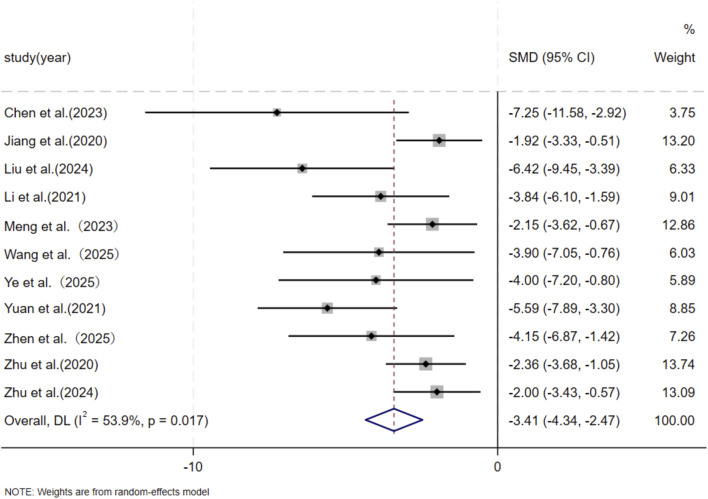
Forest plot visually illustrates the effect of exosome therapy on the expression of α-SMA. CI confidence interval, SMD standard mean difference, DL Der simonian Laird Random effects method.

**FIGURE 8:**
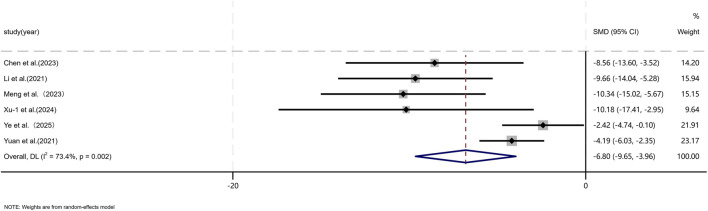
Forest plot visually illustrates the effect of exosome therapy on the expression of TGF-β1. CI confidence interval, SMD standard mean difference, DL Der simonian Laird Random effects method.

Five independent studies (Chen et al., 2023; Jiang et al., 2020; Liu et al., 2024; Li et al., 2021; Meng et al., 2024; Tian et al., 2024; Wang et al., 2025; Ye et al., 2025; Yuan et al., 2021; Zhen et al., 2025; Zhu et al., 2024; Zhu et al., 2020) comprising a total of 136 animal models, evaluated α-SMA expression, with 65 models in the experimental group and 71 in the control group. Six studies (Chen et al., 2023; Li et al., 2021; Meng et al., 2024; Xu Z. et al., 2024; Ye et al., 2025; Yuan et al., 2021) including 60 animal models equally distributed between experimental and control groups (n = 30 per group), assessed TGF-β1 expression. TGF is a master regulatory signaling pathway governing multiple fibroblast functions in scar tissue and represents the most classical pathway involved in the regulation of collagen synthesis (Bran et al., 2009; Zhang et al., 2020). In addition, the expression of α-SMA shows a striking contrast between hypertrophic scars and keloids and normal skin, with aberrant scars exhibiting pronounced α-SMA expression, whereas normal skin displays minimal or absent expression (Limandjaja et al., 2020). Given the substantial heterogeneity among studies (*p* < 0.05, I^2^ > 50%), a DerSimonian–Laird random-effects model was applied. At the final assessment time point, pooled analysis demonstrated a significant reduction in α-SMA expression in the MSC-EV–treated group compared with controls (SMD = −3.41, 95% CI: −4.34 to −2.47, p = 0.017) (Figure 7). Similarly, TGF-β1 expression was significantly lower in the experimental group than in the control group (SMD = −6.80, 95% CI: −9.65 to −3.96, *p* < 0.001) (Figure 8).

##### 3.4.2.4 Assessment of risk of bias

**FIGURE 9:**
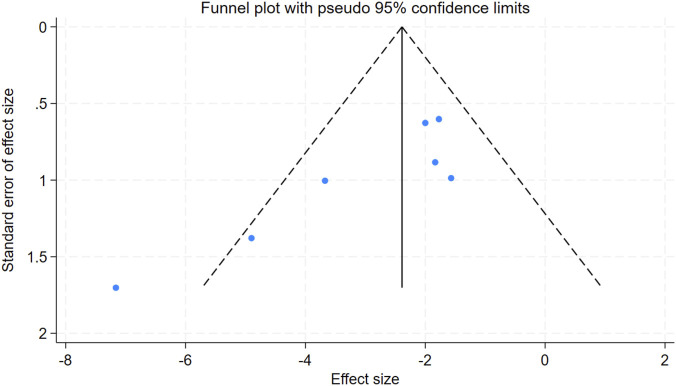
Funnel plot for the scar reduction rate. SMD standard mean difference.

Publication bias was evaluated for scar size metrics across the seven studies included in the analysis. Subsequent assessment using Egger’s test yielded a p-value of 0.018 for the scar reduction rate (P < 0.05), suggesting the presence of potential publication bias (Figure 9). To address this, the trim-and-fill method was employed, which identified no missing studies. Although the Egger test indicated asymmetry in the funnel plot, the trim-and-fill procedure did not identify any studies that would require supplementation. This implies that the existing results are likely to be minimally influenced by publication bias, thus supporting the robustness of the conclusions. The observed funnel plot asymmetry is more likely to be due to methodological heterogeneity across the studies rather than publication bias.

### 3.5 Sensitivity analysis

Sensitivity analysis of scar reduction rate, collagen deposition, the expression of TGF-β1 and α-SMA, and fibroblast migration and proliferation was performed. No individual study’s effect size lay beyond the 95% confidence interval, nor did it overturn the pooled estimate, confirming that no single investigation exerted a disproportionate influence on the overall results; thus, the meta-analytic findings for every outcome measure remain robust.

## 4 Discussion

### 4.1 Summary of main findings

Hypertrophic scars and keloids are fibroproliferative skin disorders characterized by excessive fibroblast activity. These conditions cause significant functional and cosmetic impairments, which contribute to a substantial psychological burden for affected individuals (Frech et al., 2023). Despite the availability of various therapeutic options, the treatment of hypertrophic scars and keloids remains challenging. No single modality is universally recommended, and none provide complete therapeutic efficacy (Tripathi et al., 2020). This review focuses on stem cell-derived exosome therapy, a novel cell-free treatment approach that has recently emerged as a promising strategy for intervention. Exosomes, due to their natural origin, exceptional biocompatibility, and multifunctional properties, are increasingly recognized as innovative platforms for drug delivery, immune modulation, and precision-based therapies (Zhou et al., 2023). Exosomes have the ability to regulate nearly all stages of wound healing and keloid suppression, including immune and inflammatory modulation, macrophage polarization, angiogenesis, cellular proliferation and migration, and ECM remodeling (Ma et al., 2022; Zhou et al., 2023; Ti et al., 2015; Shabbir et al., 2015). Achieving scar-free healing during the early stages of wound repair is the ideal strategy for minimizing scar formation. However, chronic wounds, particularly those with increasing prevalence in younger populations (<65 years), remain a significant public health concern, underscoring the ongoing relevance of this research (Sen, 2023; Carter et al., 2023). The transition from wound clot to granulation tissue in hypertrophic scars and keloids is regulated by a delicate balance between ECM protein deposition and degradation. Disruption of this process leads to abnormal scarring, ultimately resulting in the development of either keloids or hypertrophic scars (Tripathi et al., 2020). The transient transformation of fibroblasts into myofibroblasts is essential for acute wound healing; however, prolonged activation of myofibroblasts contributes to scar formation and fibrosis. (Younesi et al., 2024). Dysregulation of TGF-β/Smad signaling is a major contributor to scar formation and fibrosis, as it triggers excessive ECM deposition as well as the generation and activation of local myofibroblasts. This process is characterized by aberrant collagen synthesis and accumulation, an increased COL I/III ratio, and the formation of abnormally cross-linked collagen fiber bundles, ultimately driving the phenotypic transition of fibroblasts into myofibroblasts (Jiang et al., 2023; Zhang et al., 2020; Lichtman et al., 2016; Tang et al., 2018). During the wound-healing phase, activation of myofibroblasts promotes wound closure through ECM production and contraction. As myofibroblasts further mature, the incorporation of α-SMA, a specific actin isoform, not only enhances fibroblast contractility but also reinforces intracellular mechanotransduction feedback loops that sustain myofibroblast activation, leading to the development of excessively proliferative and rigid scars. Consequently, α-SMA is widely regarded as the most commonly used marker for identifying myofibroblasts in both cell cultures and fibrotic tissue sections Understanding Keloid Pathobiology (Skalli et al., 1986; Younesi et al., 2024; Hinz, 2016; Hinz et al., 2001; Talele et al., 2015). In summary, cutaneous wound healing involves the dynamic formation and remodeling of the collagen matrix by fibroblasts and myofibroblasts over time (Tan et al., 2019). Accordingly, the present study considers TGF-β and α-SMA as key regulatory targets through which MSC-EVs modulate scar formation.

Recent studies have increasingly focused on exosome-based therapies for wound healing. These analyses suggest that exosomes may attenuate the expression of genes involved in ECM remodeling, which is typically associated with scar formation. In the early phases of wound healing, there is an accelerated deposition and maturation of collagen. However, during the remodeling phase, the ECM and granulation tissue undergo dynamic restructuring, which involves both degradation and resynthesis processes, playing pivotal roles in the progression of Regulate the process of scar formation (Yuan T. et al., 2023). Our analysis highlights the primary role of exosome-mediated effects during the proliferative and remodeling stages of healing, while also acknowledging the influence of the initial inflammatory phase on fibrosis. Mast cells and macrophages are key players in this process. Mast cells contribute to fibrosis by releasing chemical mediators during degranulation, which activate multiple distinct signaling pathways (Nakajima et al., 2022; Yeo et al., 2024; Siddhuraj et al., 2022). M2 macrophages are particularly important in the pathogenesis of fibrotic disorders, emphasizing the need for further animal studies to explore the therapeutic potential of MSC-Exos interventions during the inflammatory phase (Ge et al., 2024; Dai et al., 2024).

Recent evidence increasingly supports the role of exosomes in alleviating organ fibrosis through various mechanisms. Deng demonstrated that in a myocardial infarction model, exosomes derived from ADSCs mitigated cardiac damage by activating the S1P/SK1/S1PR1 signaling pathway and promoting M2 macrophage polarization (Deng et al., 2019). Shi observed that in a bleomycin-induced idiopathic pulmonary fibrosis model, exosomes derived from human umbilical cord mesenchymal stem cells extracellular vesicles (hucMSC-EVs) alleviated fibrosis by delivering miR-21-5p and miR-23-3p. These microRNAs inhibited TGF-β signaling, leading to a reduction in myofibroblast differentiation and collagen deposition (Shi et al., 2021). In models of silicosis-induced pulmonary fibrosis, ADSC-Exos reduced collagen deposition and slowed fibrosis progression (Bandeira et al., 2018). In a liver fibrosis model, treatment with human amniotic mesenchymal stem cell-derived EVs resulted in a reduction of α-SMA expression and fibrotic areas by inhibiting the activation of Kupffer cells and hepatic stellate cells (Ohara et al., 2018). In a renal fibrosis model, human umbilical cord mesenchymal stem cell-derived exosomes (hucMSC-Exo) attenuated collagen deposition and interstitial fibrosis by inhibiting YAP activity through CK1δ/β-TRANP signaling (Ji et al., 2020). Despite these promising results, the therapeutic efficacy of exosomes is somewhat limited by the inherent inefficiency of native EVs and their inability to target multiple organs effectively. To maximize their therapeutic potential, modifications to improve delivery and targeting capabilities are often required (Zhang et al., 2024).

Given the therapeutic potential of exosomes in various diseases, there has been increasing interest in the development of engineered or hybrid exosomes. However, the clinical application of exosomes is limited by several challenges, including difficulties in large-scale production, instability under varying environmental conditions, and aggregation during storage (Mondal et al., 2023). While synthetic polymers used in nanocarrier design offer enhanced stability and controlled release properties, their clinical translation is hindered by concerns regarding safety, compatibility, and toxicity (Agrawal et al., 2014; Ahmad et al., 2022). This has spurred the exploration of hybrid exosomes, which aim to combine the benefits of both exosomes and synthetic systems, potentially enhancing their clinical efficacy. In addition, plant-derived exosome-mimetic nanoparticles exhibit inherent biocompatibility, anti-inflammatory, and antioxidant properties, along with renewable and sustainable characteristics, making them promising candidates for scalable production and clinical application (Feng et al., 2024). The convergence of tissue engineering and nanotechnology has accelerated the development of extracellular vesicle-based strategies, with engineered cargo loading, targeted delivery, and stimulus-responsive systems showing substantial promise for tissue regeneration and repair. Despite these advances, exosome-nanotechnology hybrid systems remain largely confined to preclinical studies (Liu et al., 2022). Extensive clinical trials are necessary to validate their translational potential and establish their efficacy in clinical settings.

The potential of EVs in endogenous tissue remodeling and therapeutic tissue repair has been increasingly recognized, leading to a growing number of early-phase clinical trials investigating EV-based therapies. Nevertheless, substantial challenges continue to hinder their clinical translation, including compliance with current Good Manufacturing Practice (cGMP) guidelines, high reproducibility in allogeneic settings, scalability, stability, storage, and large-scale production under stringent storage and clinical quality control requirements (Elsharkasy et al., 2020).To enable the successful clinical implementation of exosomes as a promising acellular therapeutic strategy across diverse medical fields, it is imperative that researchers and manufacturers strictly adhere to cGMP standards. This entails the establishment of standardized production processes, harmonized quality control systems, and well-defined clinical research protocols to ensure safety and efficacy (Elsharkasy et al., 2020; Lener et al., 2015). Furthermore, coordinated global efforts are required to develop industry-wide standards and specialized guidelines focused on cellular activity and drug mechanisms (Li et al., 2025). Such frameworks would provide consistent reference criteria for both preclinical and clinical studies and, to some extent, mitigate methodological heterogeneity across investigations.

In conclusion, exosomes play a pivotal role in cellular signaling due to their complex composition, which includes a diverse array of signaling cargos such as proteins, lipids, surface receptors, enzymes, cytokines, transcription factors, and nucleic acids (Hade et al., 2021). This extensive cargo enables exosomes to facilitate both targeted therapy and disease diagnosis. While the majority of studies included in our analysis focused on evaluating the effects of individual components of MSC-Exos on specific skin cells, there remains a gap in systematically assessing the bioactive components of exosomes and their broader impact on various skin cell types (Zhou et al., 2023). A more comprehensive evaluation of these bioactive components is essential to fully understand their therapeutic potential and mechanisms of action.

### 4.2 Limitations

When evaluating the included studies using the SYRCLE tool, several studies failed to report the use of randomized controlled trial (RCT) methodologies, and nearly all experiments lacked detailed descriptions of specific random allocation methods. This resulted in a potential risk of bias across multiple study design domains. Furthermore, the limited number of studies eligible for meta-analysis was insufficient to adequately assess publication bias. Regarding study design, there was considerable variability in the animal models used, wound creation techniques, wound diameter or area measurements, and methods for assessing scar size, which may have impaired the precise evaluation of study outcomes. In addition, interspecies differences in anatomy and physiology between animal models and humans may substantially influence experimental outcomes. For example, wound healing in rodents occurs predominantly through wound contraction, whereas rabbits can partially recapitulate human metabolic characteristics (Gottrup et al., 2000; Abdullahi et al., 2014), and porcine skin morphology, physiological structure, and wound-healing processes more closely resemble those of human skin (Sullivan et al., 2001). In recent years, to enhance the translational relevance of basic research to clinical applications, increasingly sophisticated *in vitro* human skin–mimicking models have been developed, ranging from conventional two-dimensional (2D) skin cell cultures to three-dimensional (3D) tissue-engineered constructs. These models more closely approximate native human skin architecture and are able to capture key features of scar formation to a certain extent (Hofmann et al., 2023).

## 5 Conclusion

Our findings suggest that MSC-EVs exert therapeutic effects on the formation of hypertrophic scars and keloids, resulting in noticeable scar reduction through several mechanisms. These include the attenuation of collagen deposition, the reduction of sustained myofibroblast activation, and the inhibition of fibroblast proliferation and migration. Despite the heterogeneity observed across studies, MSC-EVs consistently exhibited beneficial therapeutic outcomes, highlighting their promising potential for translation into clinical applications.
