# Supplementary material for: Therapeutic value of mesenchymal stem cell-derived extracellular vesicles in hypertrophic and keloid scars: a systematic review and meta-analysis
Source: Front Cell Dev Biol. 2026 Jan 28;14:1739106. doi: 10.3389/fcell.2026.1739106 (PMC12891142; doi:10.3389/fcell.2026.1739106)
Supplement: Supplementary file 1 [file DataSheet1.docx]

# Therapeutic value of Mesenchymal stem cell-derived extracellular vesicles in Hypertrophic and Keloid Scars: A systematic review and meta-analysis

Search strategy

Database：Pubmed

Data searched: 29 August, 2025

Records retrieved:122

((((((((((Mesenchymal Stromal Cells[MeSH Terms]) OR (Mesenchymal Stem Cell Transplantation[MeSH Terms])) OR (Multipotent Stem Cells[MeSH Terms])) OR (MSC[Title/Abstract] OR MSCs[Title/Abstract] OR ADMSC[Title/Abstract] OR ADMSCs[Title/Abstract] OR BM-MSC[Title/Abstract] OR BM-MSCs[Title/Abstract] OR BMD-MSC[Title/Abstract] OR BMD-MSCs[Title/Abstract] OR BMDMSC[Title/Abstract] OR BMDMSCs[Title/Abstract])) OR (MSC[Text Word] OR MSCs[Text Word] OR ADMSC[Text Word] OR ADMSCs[Text Word] OR BM-MSC[Text Word] OR BM-MSCs[Text Word] OR BMD-MSC[Text Word] OR BMD-MSCs[Text Word] OR BMDMSC[Text Word] OR BMDMSCs[Text Word])) OR ((mesenchymal[Title/Abstract] AND (cell*[Title/Abstract] OR "stem cell*"[Title/Abstract] OR "stromal cell*"[Title/Abstract] OR progenitor*[Title/Abstract] OR multipotent[Title/Abstract] OR "bone marrow"[Title/Abstract] OR adipose[Title/Abstract] OR placenta* [Title/Abstract])))) OR ((mesenchymal[Text Word] AND (cell*[Text Word] OR "stem cell*"[Text Word] OR "stromal cell*"[Text Word] OR progenitor[Text Word] OR multipotent[Text Word] OR "bone marrow"[Text Word] OR adipose[Text Word] OR placenta* [Text Word])))) OR (((multipotent[Title/Abstract] OR multi-potent [Title/Abstract]) AND ("stroma* cell*"[Title/Abstract] OR "stem cell*" [Title/Abstract]))) OR (((multipotent[Text Word] OR multi-potent [Text Word]) AND ("stroma* cell*"[Text Word] OR "stem cell*" [Text Word]))) OR ((colony-forming[Title/Abstract] AND fibroblast*[Title/Abstract])) OR ("marrow stroma* cell*"[Title/Abstract]) OR (((bone[Title/Abstract] OR "bone marrow" [Title/Abstract] OR amniotic[Title/Abstract]) AND ("mesenchymal stem cell*" [Title/Abstract]))) OR ((Adipose[Title/Abstract] OR bone[Title/Abstract] OR placenta*[Title/Abstract] OR amniotic [Title/Abstract]) AND ("Stem Cell*" [Title/Abstract])) OR ((adipose-Derived[Title/Abstract] OR bone-Derived[Title/Abstract] OR placenta*-Derived[Title/Abstract] OR amniotic-Derived[Title/Abstract]) AND "Stem Cell*"[Title/Abstract]))) AND (((((((((Exosomes[MeSH Terms]) OR (Extracellular Vesicles[MeSH Terms])) OR (Cell-Derived Microparticles[MeSH Terms])) OR ((exosom*[Title/Abstract]) OR (exosom*[Text Word]))) OR (((microvesicle*[Title/Abstract] OR "micro vesicle*" [Title/Abstract])) OR ((microvesicle*[Text Word] OR "micro vesicle*" [Text Word])))) OR (((cell[Title/Abstract] OR cells[Title/Abstract] OR extracellular [Title/Abstract]) AND vesicle* [Title/Abstract]))) OR ((microparticle*[Title/Abstract]) OR (microparticle*[Text Word]))) OR ((extracellular[Text Word] AND vesicle* [Text Word]))) OR (((cell[Text Word] OR cells [Text Word]) AND vesicle* [Text Word])))) AND ((((((Keloid[Title/Abstract]) OR (Hypertrophic[Title/Abstract] AND (Scar*[Title/Abstract] OR Cicatri* [Title/Abstract]))) OR ("hypertrophic scar"[Title/Abstract] AND (formation[Title/Abstract] OR tissue[Title/Abstract] OR contracture [Title/Abstract]))) OR (pathologic*[Title/Abstract] AND (scar*[Title/Abstract] OR cicatrix[Title/Abstract]))) OR ((scar*[Title/Abstract] OR keloid*[Title/Abstract]) AND fibrosis[Title/Abstract])) OR ((Abnormal[Title/Abstract] OR Excessive[Title/Abstract])AND scar*[Title/Abstract]))

Database：Web of Science

Data searched: 29 August, 2025

Records retrieved: 19

TI=(mesenchymal stroma* cell*) OR TI=((mesenchymal and (cell* or stem or stromal or progenitor or multipotent or 'bone marrow' or adipose or placenta*))) OR TI=("marrow stroma* cell*") and Preprint Citation Index (Exclude – Database) OR TI=(((adipose-Derived or bone-Derived or placenta*-Derived or amniotic-Derived) and 'Stem Cell*'))

OR TI=(((adipose or bone or placenta* or amniotic) and 'stem cells')) OR TI=(((bone or "bone marrow" or amniotic) and "'mesenchymal stem cell*")) OR TI=(msc or mscs or admsc or admscs or 'bm msc' or 'bm mscs' or 'bmd msc' or 'bmd mscs' or bmdmsc or bmdmscs) OR TI=((multipotent or "multi potent") and ("stroma* cell*" or "stem cell*"))

AND TI=(exosom*) OR TI=(membrane microparticle*) OR TI=((microvesicle* or micro) and vesicle*) OR TI=(cell* and vesicle*) OR TI=(extracellular and vesicle*) OR TI=(microparticle*) OR TI=((cell* or extracellular) and vesicle*) AND TI=(keloid*) OR TI=(hypertrophic scar*) OR TI=((keloid* or hypertrophic) and (scar* or cicatri*)) OR TI=('hypertrophic scar' and (formation or tissue or contracture)) OR TI=(pathologic* and (scar* or cicatrix)) OR TI=((scar* or keloid) and fibrosis) OR TI=((abnormal or excessive) and scar*)

Database：Ovid MEDLINE Complete

Data searched: 29 August, 2025

Records retrieved: 96

mesenchymal stroma* cell*.ab,bt,kf. OR (msc or mscs or admsc or admscs or 'bm msc' or 'bm mscs' or 'bmd msc' or 'bmd mscs' or bmdmsc or bmdmscs).ab,bt,kf. OR (mesenchymal and (cell* or stem or stromal or progenitor or multipotent or 'bone marrow' or adipose or placenta*)).ab,bt,kf. OR ((multipotent or "multi potent") and ("stroma* cell*" or "stem cell*")).ab,bt,kf. OR "marrow stroma* cell*".ab,bt,kf. OR ((adipose-Derived or bone-Derived or placenta*-Derived or amniotic-Derived) and 'Stem Cell*').ab,bt,kf. OR ((adipose or bone or placenta* or amniotic) and 'stem cells').ab,bt,kf. OR ((bone or "bone marrow" or amniotic) and "'mesenchymal stem cell*").ab,bt,kf. AND "exosom*".ab,bt,kf. OR "membrane microparticle*".ab,bt,kf. OR ((microvesicle* or micro) and vesicle*).ab,bt,kf. OR (cell* and vesicle*).ab,bt,kf. OR (extracellular and vesicle*).ab,bt,kf. OR "microparticle*".ab,bt,kf. OR ((cell* or extracellular) and vesicle*).ab,bt,kf. AND "keloid*".ab,bt,kf. OR hypertrophic scar*.ab,bt,kf. OR ((keloid* or hypertrophic) and (scar* or cicatri*)).ab,bt,kf. OR ('hypertrophic scar' and (formation or tissue or contracture)).ab,bt,kf. OR (pathologic* and (scar* or cicatrix)).ab,bt,kf. OR ((scar* or keloid) and fibrosis).ab,bt,kf. OR ((abnormal or excessive) and scar*).ab,bt,kf.

Database：Embase

Data searched: 29 August, 2025

Records retrieved:155

(abnormal:ti,ab OR excessive:ti,ab) AND scar*:ti,ab OR (scar*:ti,ab OR keloid:ti,ab) AND fibrosis:ti,ab OR pathologic*:ti,ab AND (scar*:ti,ab OR cicatrix:ti,ab) OR 'hypertrophic scar':ti,ab AND (formation:ti,ab OR tissue:ti,ab OR contracture:ti,ab) OR (keloid:ti,ab OR hypertrophic:ti,ab) AND (scar*:ti,ab OR cicatri*:ti,ab) OR 'hypertrophic scar'/exp OR 'keloid'/exp AND cell* AND vesicle*:kw,ti,ab OR extracellular:kw,ti,ab AND vesicle*:kw,ti,ab OR microparticle*:kw,ti,ab OR ((cell* OR extracellular) NEAR/2 vesicle*):ab,ti OR (microvesicle*:kw,ab,ti OR micro:kw,ab,ti) AND vesicle*:kw,ab,ti OR 'membrane microparticle'/exp OR 'membrane microparticle' OR exosom*:kw,ab,ti OR 'exosome'/exp OR 'exosome' AND adipose-Derived:ti,ab OR bone-Derived:ti,ab OR placenta*-Derived:ti,ab OR amniotic-Derived:ti,ab AND 'Stem Cell*':ti,ab OR (adipose:ab,ti OR bone:ab,ti OR placenta*:ab,ti OR amniotic:ab,ti) AND 'stem cells':ab,ti OR (bone:ab,ti OR 'bone marrow':ab,ti OR amniotic:ab,ti) AND 'mesenchymal stem cell*':ab,ti OR 'mesoderm'/de OR 'marrow stroma* cell*':ab,ti OR ('colony forming' NEAR/2 fibroblast*):ab,ti OR (multipotent:kw OR 'multi potent':kw) AND ('stroma* cell*':kw OR 'stem cell*':kw) OR ((multipotent OR 'multi potent') NEXT/1 ('stroma* cell*' OR 'stem cell*')):ab,ti OR mesenchymal:kw AND (cell*:kw OR stem:kw OR stromal:kw OR progenitor:kw OR multipotent:kw OR 'bone marrow':kw OR adipose:kw OR placenta*:kw) OR (mesenchymal NEAR/5 (cell* OR stem OR stromal OR progenitor* OR multipotent OR 'bone marrow' OR adipose OR placenta*)):ab,ti OR msc:ab,ti OR mscs:ab,ti OR admsc:ab,ti OR admscs:ab,ti OR 'bm msc':ab,ti OR 'bm mscs':ab,ti OR 'bmd msc':ab,ti OR 'bmd mscs':ab,ti OR bmdmsc:ab,ti OR bmdmscs:ab,ti

'multipotent stem cell'/exp OR 'mesenchymal stem cell transplantation'/exp OR 'mesenchymal stroma cell'/exp

Database：Cochrane

Data searched: 29 August, 2025

Records retrieved: 11

MeSH descriptor: [Mesenchymal Stem Cells] explode all trees OR MeSH descriptor: [Mesenchymal Stem Cell Transplantation] explode all trees OR MeSH descriptor: [Multipotent Stem Cells] explode all trees OR MSC:ti,ab,kw OR MSCs:ti,ab,kw OR ADMSC:ti,ab,kw OR ADMSCs:ti,ab,kw OR BM-MSC:ti,ab,kw OR BM-MSCs:ti,ab,kw OR BMD-MSC:ti,ab,kw OR BMD-MSCs:ti,ab,kw OR BMDMSC:ti,ab,kw OR BMDMSCs:ti,ab,kw OR (mesenchymal AND (cell* OR stem OR stromal OR progenitor* OR multipotent OR "bone marrow" OR adipose OR placenta* )):ti,ab,kw OR ((multipotent:ti,ab,kw) NEXT ((stroma* NEXT cell*):ti,ab,kw OR ("stem" NEXT cell*):ti,ab,kw)) OR ((multipotent OR multi-potent ) AND ((stroma* NEXT cell*) OR ("stem" NEXT cell*))):ti,ab,kw OR (colony-forming:ti,ab NEAR/2 fibroblast*:ti,ab) OR marrow stroma* cell*:ti,ab,kw OR (adipose:ab,ti OR bone:ab,ti OR placenta*:ab,ti OR amniotic:ab,ti) AND 'stem cell*':ab,ti OR (bone:ab,ti OR 'bone marrow':ab,ti OR amniotic:ab,ti) AND 'mesenchymal stem cell*':ab,ti OR (adipose Derived:ab,ti OR bone Derived:ab,ti OR placenta* Derived:ab,ti OR amniotic Derived:ab,ti) AND 'Stem Cell*':ab,ti AND MeSH descriptor: [Exosomes] explode all trees OR MeSH descriptor: [Extracellular Vesicles] explode all trees OR MeSH descriptor: [Cell-Derived Microparticles] explode all trees OR exosom*:kw,ti,ab OR microvesicle*:ti,ab,kw OR ("micro" NEXT vesicle*):ti,ab,kw OR (cell:ti,ab OR cells:ti,ab OR extracellular:ti,ab) NEAR/2 vesicle*:ti,ab OR microparticle*:kw,ti,ab OR extracellular:kw AND vesicle*:kw OR ((cell or cells) and vesicle*):kw AND MeSH descriptor: [Keloid] explode all trees OR MeSH descriptor: [Cicatrix, Hypertrophic] explode all trees OR 'hypertrophic scar':ti,ab AND (formation:ti,ab OR tissue:ti,ab OR contracture:ti,ab) OR (keloid*:ti,ab OR hypertrophic:ti,ab) AND (scar*:ti,ab OR cicatri*:ti,ab) OR keloid:ti,ab,kw OR "hypertrophic scar":ti,ab,kw OR pathologic*:ti,ab AND (scar*:ti,ab OR cicatrix:ti,ab) OR (scar*:ti,ab OR keloid:ti,ab) AND fibrosis:ti,ab OR (abnormal:ti,ab OR excessive:ti,ab) AND scar*:ti,ab OR scar*:ti,ab,kw

Database：CNKI

Data searched: 29 August, 2025

Records retrieved:20

Title:Mesenchymal Stem Cells or Mesenchymal Stromal Cells or derived stem cell

and Title:Exosomes or Extracellular Vesicles and Title:Keloid or Hypertrophic scar or pathological scar or abnormal scar

Database：Wanfang

Data searched: 29 August, 2025

Records retrieved:24

Title:(mesenchymal (stem OR stromal) cells OR Derived stem cell) and Title:(keloids OR Hypertrophic scars OR pathological scar OR abnormal scar) and Title:(exosomes OR Extracellular Vesicles)
